# Machine Learning-Based Software for Predicting *Pseudomonas* spp. Growth Dynamics in Culture Media

**DOI:** 10.3390/life14111490

**Published:** 2024-11-15

**Authors:** Fatih Tarlak

**Affiliations:** Department of Bioengineering, Gebze Technical University, Gebze 41400, Kocaeli, Turkey; ftarlak@gtu.edu.tr

**Keywords:** software development, *Pseudomonas* spp., machine learning, traditional modelling

## Abstract

In predictive microbiology, both primary and secondary models are widely used to estimate microbial growth, often applied through two-step or one-step modelling approaches. This study focused on developing a tool to predict the growth of *Pseudomonas* spp., a prominent bacterial genus in food spoilage, by applying machine learning regression models, including Support Vector Regression (SVR), Random Forest Regression (RFR) and Gaussian Process Regression (GPR). The key environmental factors—temperature, water activity, and pH—served as predictor variables to model the growth of *Pseudomonas* spp. in culture media. To assess model performance, these machine learning approaches were compared with traditional models, namely the Gompertz, Logistic, Baranyi, and Huang models, using statistical indicators such as the adjusted coefficient of determination (R^2^_adj_) and root mean square error (RMSE). Machine learning models provided superior accuracy over traditional approaches, with R^2^_adj_ values from 0.834 to 0.959 and RMSE values between 0.005 and 0.010, showcasing their ability to handle complex growth patterns more effectively. GPR emerged as the most accurate model for both training and testing datasets. In external validation, additional statistical indices (bias factor, *B*_f_: 0.998 to 1.047; accuracy factor, *A*_f_: 1.100 to 1.167) further supported GPR as a reliable alternative for microbial growth prediction. This machine learning-driven approach bypasses the need for the secondary modelling step required in traditional methods, highlighting its potential as a robust tool in predictive microbiology.

## 1. Introduction

Predictive food microbiology is a theoretical field within food microbiology that focuses on developing statistical models to forecast microbial behaviour in food environments by merging traditional microbiological knowledge with mathematical and statistical principles [1]. While the use of predictive models dates back to the early 20th century, advancements in computer technology have significantly accelerated the progress of predictive microbiology in the 21st century. These models are utilized to determine conditions within food environments that mitigate or delay the adverse effects of microbial contamination. In traditional predictive microbiology, mathematical models are generally categorized into two types: primary and secondary models [2]. Primary models describe the behaviour of microorganisms over time under static environmental conditions, essentially capturing how microbial populations grow, survive, or die when external factors remain constant. Secondary models, in contrast, account for the influence of environmental variables—such as temperature, pH, and water activity—and food matrices on the parameters of the primary models. While this conventional modelling framework is often effective in predicting microbial behaviour, it does have certain limitations. A significant concern is the potential accumulation and amplification of errors, which can occur because the nonlinear regression process is performed twice—once in developing the primary model and again when integrating environmental factors in the secondary model [3,4,5].

In recent years, the application of machine learning algorithms has gained significant momentum across various research fields. This surge is largely driven by three key technological advancements: first, the ability to quickly capture vast amounts of digital data; second, the exponential growth in affordable computing power and data storage; and third, the development of a global network enabling rapid data transfer. Numerous studies have explored the use of machine learning (ML) techniques in food safety and modelling [6,7,8,9]. Machine learning methods are particularly effective in identifying underlying relationships between explanatory and response variables in datasets, making ML-based regression approaches capable of predicting population behaviours and enhancing the predictive accuracy of bacterial growth patterns. Despite these promising advancements, the application of machine learning algorithms to predict microbial behaviour in food systems remains relatively uncommon. Furthermore, to the best of our knowledge, no studies have yet compared traditional modelling approaches with machine learning models within the field of predictive microbiology.

Both traditional modelling techniques and machine learning approaches can be utilized to predict microbial behaviour and estimate the shelf life of food products [10]. Traditional models rely on predefined mathematical equations and structured computational methods, while machine learning techniques leverage algorithms to uncover patterns and generate predictions directly from data [11]. Machine learning offers a significant advantage in its ability to capture complex, nonlinear relationships, making it particularly useful for analyzing large, diverse datasets. However, it often requires substantial amounts of training data and can pose interpretability challenges. Traditional models, on the other hand, are generally grounded in established biological and chemical principles, which makes them easier to interpret. These models may be preferable when data availability is limited or when there is a need for a straightforward, transparent explanation of results [12].

The primary objective of this work is to develop software that utilizes machine learning-based regression methods—specifically Support Vector Regression (SVR), Random Forest Regression (RFR) and Gaussian Process Regression (GPR)—to predict and quantify the behaviour of *Pseudomonas* spp. in culture media. Temperature, water activity, and pH were the key predictor variables used to estimate microbial growth. The performance of these machine learning models was assessed by comparing them to traditional models, such as the modified Gompertz, Logistic, Baranyi, and Huang models, using statistical metrics like the adjusted coefficient of determination (R^2^_adj_) and root mean square error (RMSE).

## 2. Material and Methods

**Figure 1:**
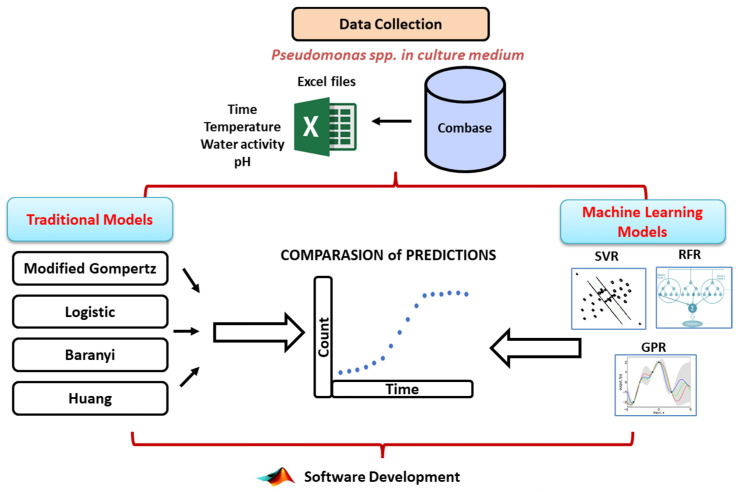
A flow chart outlining the main steps followed in the present study.

This study consists of five primary steps shown in Figure 1: (i) bacterial data for *Pseudomonas* spp. in culture mediums was gathered from the ComBase database in Excel format, (ii) traditional modelling was conducted, (iii) a range of machine learning-based regression models—such as Support Vector Regression, Gaussian Process Regression, Random Forest Regression, and decision tree regression—were applied, (iv) a comparative analysis of the performance between traditional and machine learning models was performed, and (v) the validation phase and software development were finalized. Further details on each of these five steps are provided in the subsequent sections.

### 2.1. Data Gathering

The ComBase database (www.combase.cc) hosts around 60,000 bacterial datasets sourced from research organizations and scientific publications. Each dataset includes specific features and environmental conditions—such as food category, food name, temperature, pH, water activity, conditions, and time—allowing for a detailed classification of microbial factors and responses. For this study, 2422 bacterial data points describing the growth behaviour of *Pseudomonas* spp. in culture medium were extracted from ComBase, along with detailed individual information on time, temperature, water activity, and pH, and compiled into Excel files.

### 2.2. Modelling

Multicollinearity among independent variables can inflate the variance of regression coefficients, potentially leading to unstable models and reduced prediction accuracy [13]. To address this, a correlation analysis was performed on the predictor variables—temperature, water activity, and pH—to assess any potential interdependence before modelling. High correlations between predictors can signal multicollinearity, which can compromise model reliability and accuracy. As shown in the correlation values (Table 1), there were no significant relationships between the variables: the correlation between temperature and water activity was approximately −0.048, indicating a negligible negative relationship; the temperature and pH correlation was around 0.098, reflecting a very weak positive relationship, and water activity and pH had a correlation of −0.164, also suggesting a weak negative relationship. These low correlation values confirm that temperature, water activity, and pH function independently, with minimal overlap in their predictive effects. These three factors were chosen as the main predictors for modelling the growth of *Pseudomonas* spp. in the culture medium, ensuring a stable and accurate predictive model.

#### 2.2.1. Primary Models

The modified Gompertz, Logistic, Baranyi and Huang models are among the most commonly used sigmoid functions for modelling bacterial growth behaviour. Under constant environmental conditions, the modified Gompertz and Logistic models are defined by Equations (1) and (2), respectively [14]:

(1) $${x\left(t\right)=x_{0}+(x}_{max}-x_{0})\cdot exp\left\{-exp\left[\frac{r_{max}\cdot e}{{(x}_{max}-x_{0})}\cdot \left(\lambda -t\right)+1\right]\right\}$$

(2) $$x\left(t\right)=x_{0}+\frac{{(x}_{max}-x_{0})}{\left\{1+\exp\left[\frac{{4\cdot r}_{max}}{{(x}_{max}-x_{0})}\cdot \left(\lambda -t\right)+2\right]\right\}}$$

where t is the time (h), x(t) is the bacterial population concentration (log CFU/g) at time t, x_0_ is the initial bacterial population concentration (log CFU/g), x_max_ is the maximum bacterial population concentration (log CFU/g), r_max_ is the maximum bacterial growth rate (log CFU/h), and λ is the lag phase duration (h).

Other widely used primary functions include the Baranyi and Huang models, which are represented by Equations (3)–(6) [15,16]:

(3) $$y\left(t\right)=y_{0}+{µ}_{max}F\left(t\right)-ln\left(1+\frac{e^{{µ}_{max}F\left(t\right)}-1}{e^{\left(y_{max}-y_{0}\right)}}\right)$$

(4) $$F\left(t\right)=t+\frac{1}{v}ln\left(e^{-vt}+e^{-{µ}_{max}\lambda }-e^{\left(-vt-{µ}_{max}\lambda \right)}\right)$$

(5) $$y\left(t\right)=y_{0}+y_{max}-ln(e^{y_{0}}+\left[e^{y_{max}}-e^{y_{0}}\right]\cdot e^{{-µ}_{max}B(t)})$$

(6) $$B\left(t\right)=t+\frac{1}{4}ln\left(\frac{1+e^{-4(t-\lambda )}}{1+e^{4\lambda }}\right)$$

where t is the time (h), y(t) is the bacterial population concentration (ln CFU/g) at time t, y_0_ is the initial bacterial population concentration (ln CFU/g), y_max_ is the maximum bacterial population concentration (ln CFU/g), µ_max_ is the maximum specific bacterial growth rate (1/h), λ is the lag phase duration (h), and v is the rate of increase of the limiting substrate, assumed to be equal to µ_max_.

Since the primary models use different scales to count microbe populations, the growth rate values (r_max_) obtained from the Modified Gompertz and Logistic models are converted to maximum specific growth rate values (µ_max_) after fitting. This conversion is carried out by multiplying by ln(10) [17].

#### 2.2.2. Secondary Models

Secondary models are employed to describe the effects of various environmental factors—such as water activity, acidity and temperature—on the parameters of primary models [17]. Typically applied after fitting growth data to primary models, secondary models help clarify how factors like water activity [18], acidity and temperature influence growth rates, which is essential for managing food preservation, safety and quality [19,20,21]. The Ratkowsky model (Equation (7)), specifically, is used to detail the relationship between extrinsic factors and the maximum specific growth rate [22].

(7) $${{µ}_{max}=b_{1}\left(T-T_{0}\right)}^{2}\times b_{2}\left(a_{w}-a_{w\_min}\right)\times b_{3}\left(pH-{pH}_{min}\right)$$

where µ_max_ is the maximum specific growth rate (1/h) obtained from the primary model, a_w_ is the water activity, and a_w_min_ is the minimal water activity at which growth stops. pH_min_ is the minimal pH, and pH_max_ is the maximal pH at which growth stops. *T* is the temperature (°C), *T*_0_ is the theoretical minimum temperature (°C) for microbial growth, and *b*_1_, *b*_2_ and *b*_3_ are the regression coefficients.

Additionally, *λ* (lag phase duration) was defined as a function of µ_max_ with respect to temperature using Equation (8):

(8) $$\lambda =\frac{b_{4}}{{µ}_{max\;}(T,a_{w},\;pH)\;\;}$$

where *b*_4_ is the regression coefficient, and µ_max_ (*T*, $a_{w}$, pH) is a function of temperature, water activity and acidity, which leads *λ* to be defined as a function of storage temperature, water activity and acidity.

#### 2.2.3. Machine Learning Models

The predictive ability of each machine learning model differs based on the levels of bias and variance in the data. Support Vector Machines (SVMs) are capable of mapping data into higher-dimensional spaces to reveal both linear and nonlinear associations between predictor variables and the response. Frequently applied in both classification and regression tasks, the SVM is a nonparametric approach that uses kernel functions to define its feature space for data regression [23]. In this study, Support Vector Regression (SVR) was implemented with a radial basis function (RBF) kernel. While SVR performs well in high-dimensional settings, its effectiveness can be impacted by noise within the dataset.

Random Forest Regression (RFR) is an ensemble learning method that builds multiple decision trees on different subsets of data to improve prediction accuracy and robustness [6]. By creating numerous trees, each trained on a random sample of data points and features, RFR reduces the risk of overfitting that individual decision trees often face [23]. During prediction, each tree provides an output, and the final result is typically the average of these outputs, which enhances generalizability and reduces variance. This method’s reliance on multiple “weak learners” allows it to effectively handle complex, high-dimensional datasets with noise. RFR is known for its resilience to overfitting and ability to handle both categorical and continuous data, making it highly effective for regression tasks across various domains.

Gaussian Process Regression (GPR) is a nonlinear, non-parametric Bayesian method known for its flexibility and fully probabilistic nature [24]. Based on Gaussian distribution, GPR extends the concept of normal distributions to an infinite-dimensional, multivariate form, allowing it to model data with a high degree of adaptability. GPR constructs objective functions by measuring the distance between the estimated output probability density function (pdf) and the data, maintaining high certainty even in un-sampled regions far from the training data. However, as a non-parametric approach, GPR requires the entire training dataset for each prediction, leading to significant computational costs. The squared exponential kernel was utilized for GPR, allowing it to capture smooth, continuous patterns. Gaussian processes provide the best linear unbiased predictions at un-sampled points, offering reliable estimation in spatially sparse areas.

In machine learning, model validation commonly relies on k-fold cross-validation and hold-out validation methods [25]. In hold-out validation, the data are divided into a training set and a separate test set; the model is trained on the training set and then evaluated using the test set to gauge its performance. For k-fold cross-validation, the dataset is split into k equal parts (folds), where each fold takes a turn as the test set while the remaining folds are used for training. This process is repeated k times, and the combined results from each iteration provide a more comprehensive evaluation of model accuracy. Unlike hold-out validation, which may introduce bias due to reliance on a single split of data, k-fold cross-validation offers a more reliable performance assessment. Thus, a 10-fold cross-validation approach was chosen for this study.

### 2.3. Comparison of the Goodness of Fit

A comparison of the performance of the models was carried out by using the root mean square error (*RMSE*) and adjusted coefficient of determination (R^2^_adj_) using Equations (9) and (10), respectively:

(9) $$RMSE=\sqrt{\sum_{i=1}^{n}\frac{{\left(x_{obs}-x_{fit}\right)}^{2}}{n-s}}$$

(10) $$R_{adj}^{2}=1-\left(\frac{n-1}{n-s}\right)\left(\frac{SSE}{SST}\right)$$

where *x_obs_* is the experimental bacterial growth, *x_fit_* is the fitted value, n is the number of experiments, s is the number of parameters of the model, *SSE* is the sum of squares of errors and *SST* is the total sum of squares.

### 2.4. The Models’ Validation

Model validation is the process of assessing the predictive power of constructed models using either previously published or newly generated data. The predictive accuracy of these models can be evaluated by examining the growth kinetics of the microbes. The bias factor (*B*_f_) and accuracy factor (*A*_f_) for each global model, which provide insights into the models’ prediction ability, are outlined in Equations (11) and (12), respectively, following the approach [26].

(11) $$B_{f}=10^{\frac{\sum_{i=1}^{n}\log\left(\frac{x_{pred}}{x_{obs}}\right)}{n}}$$

(12) $$A_{f}=10^{\frac{\sum_{i=1}^{n}\left|log(x_{pred}/x_{obs})\right|}{n}}$$

where *x_pred_* denotes projected maximum values (1/h) and (h), *x_obs_* denotes experimental µ_max_ (1/h) and *λ* (h), and n is the number of experimental growth data.

The acceptable prediction zone (APZ) approach is a useful method for evaluating the overall validation performance of various predictive models. In the APZ framework, a prediction is deemed acceptable when the residual (difference between observed and predicted values) falls within an APZ range of −1 log CFU/g (fail-safe) to 0.5 log CFU/g (fail-dangerous).

To visually display validation results, prediction software was created in this study using both traditional and machine learning-based regression models. All procedures were conducted using MATLAB version 9.10.0.1710957 (R2021a) (MathWorks Inc., Natick, MA, USA) (see Supplementary Video S1).

## 3. Results and Discussion

**Figure 2:**
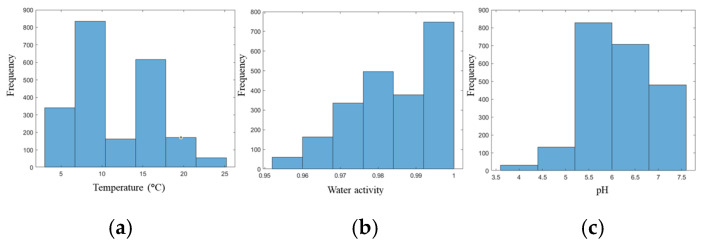
Histograms of the variables for (**a**) temperature (°C), (**b**) water activity and (**c**) pH.

The growth data points of *Pseudomonas* spp. in culture mediums collected from the ComBase database were stored with the following information: record ID, temperature (°C), water activity, pH, initial microbial population (yes/no) and time (h). The data frequency of the collected data categorized into each of the features is shown in Figure 2.

The maximum specific growth rate and lag phase duration, key growth kinetic parameters, can be modelled in relation to environmental factors such as temperature, water activity and pH. Among these, temperature plays a crucial role in influencing microbial growth behaviour in food products, as noted by [27]. In this study, the temperature range considered was 5 to 25 °C, reflecting typical conditions encountered by food products during storage, transport and retail. This range includes refrigeration temperatures (around 5–10 °C), which slow microbial growth, as well as warmer conditions up to 25 °C, where microbial activity accelerates, potentially impacting shelf life and safety. Water activity, another essential factor in microbial growth, represents the ratio between the vapour pressure of the food and the vapour pressure of distilled water in identical conditions. Most foods have a water activity level above 0.95, which is sufficient to support microbial growth, as free water is available for cellular processes. In this study, the water activity range was from 0.954 to 0.997, indicating conditions that provide ample moisture to promote microbial growth in fresh and perishable foods. The pH level of food also directly affects microbial growth by influencing enzyme activity and cellular function. In this study, pH values ranged from 4.01 to 7.40 for the culture medium, encompassing both acidic and near-neutral conditions. Acidic environments (pH around 4) inhibit many spoilage organisms, while near-neutral pH conditions (closer to 7) support a broader range of bacterial growth, potentially accelerating spoilage. These environmental factors—temperature, water activity and pH—collectively influence μ_max_ and *λ*, making them critical for predicting microbial behaviour and developing effective storage and preservation strategies in the food industry.

For model comparison, 80% of the data were allocated for training and 20% for testing. Table 2 presents the performance differences between traditional and machine learning models in microbial growth modelling during the training process. Traditional models, such as Gompertz, Logistic, Baranyi and Huang, are frequently used due to their interpretability and effectiveness in capturing standard S-shaped microbial growth patterns. These traditional models, however, rely on fixed growth structures, which restricts their flexibility in capturing complex, nonlinear growth dynamics. Although both two-step and one-step modelling approaches were initially applied, the two-step approach did not successfully fit the data across any of the traditional models. Consequently, only the results from the one-step modelling approach are presented here as the traditional modelling outcome.

**Figure 3:**
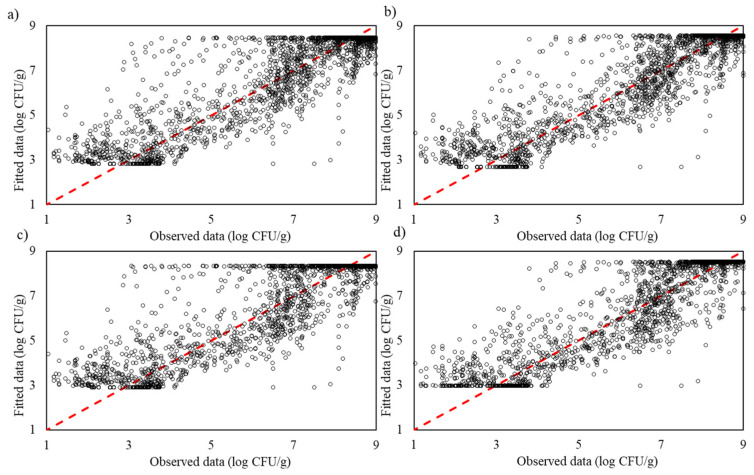
The observed and predicted *Pseudomonas* spp. in culture medium using traditional models: (**a**) modified Gompertz, (**b**) Logistic, (**c**) Baranyi and (**d**) Huang for training process.

The Gompertz model achieved an R^2^_adj_ of 0.813 and an RMSE of 0.022, indicating moderate predictive accuracy but limited flexibility. The Logistic model performed slightly better, with an R^2^_adj_ of 0.844 and an RMSE of 0.020, capturing microbial growth dynamics more effectively. Despite incorporating a lag phase, the Baranyi model had the lowest R^2^_adj_ among the traditional models (0.790) and an RMSE of 0.023, reflecting its challenges in handling complex growth behaviours. The Huang model was the most accurate among traditional models, achieving an R^2^_adj_ of 0.850 and an RMSE of 0.020, though it was still surpassed by machine learning models (Figure 3).

**Figure 4:**
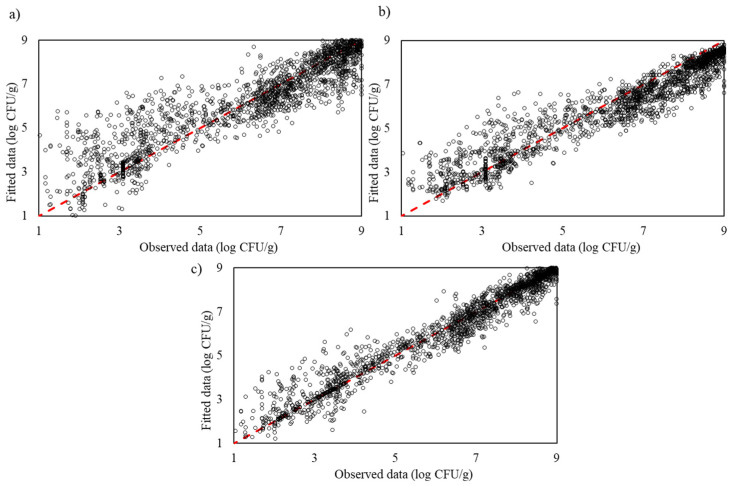
The observed and predicted *Pseudomonas* spp. in culture medium using machine learning models (**a**) Support Vector Regression, (**b**) Random Forest Regression and (**c**) Gaussian Process Regression for training process.

Machine learning models, which do not rely on predefined relationships, demonstrated greater adaptability. SVR yielded an R^2^_adj_ of 0.854 and an RMSE of 0.019, indicating solid predictive performance, though it was slightly less effective than GPR and RFR, likely due to sensitivity in parameter tuning. Random Forest Regression achieved an R^2^_adj_ of 0.893 and an RMSE of 0.017, benefiting from its ensemble approach, which captures complex interactions between variables. Gaussian Process Regression provided the highest R^2^_adj_ (0.959) and the lowest RMSE (0.010), showcasing exceptional accuracy and robustness in modelling nonlinear growth patterns. These results illustrate that while traditional models like Huang offer reasonable accuracy, machine learning models, particularly GPR, deliver superior predictive performance and are better suited for modelling complex microbial growth dynamics during the training process (Figure 4).

**Figure 5:**
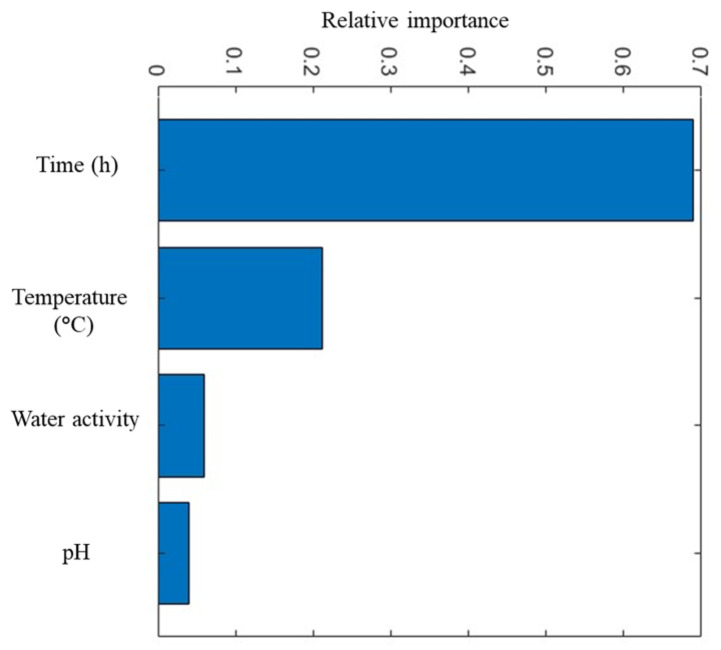
The relative importance of predictor variables to microorganism populations in culture medium using Gaussian Process Regression.

The bar chart highlights the relative importance of four predictors—time, temperature, water activity and pH—in modelling the growth of *Pseudomonas* spp. (Figure 5). The results show that time is by far the most influential factor in predicting *Pseudomonas* growth, indicating that microbial growth patterns are significantly dependent on the duration of exposure under given conditions. This aligns with biological expectations, as microbial populations typically increase exponentially over time when other growth conditions remain constant. Temperature is the second most significant factor. This reflects the sensitivity of *Pseudomonas* growth rates to temperature changes, as temperature is known to play a critical role in enzymatic activity and cellular processes. Higher or optimal temperatures generally accelerate microbial growth until a threshold, beyond which growth rates decline. Therefore, temperature control is essential in limiting *Pseudomonas* growth, especially in food storage and handling. Water activity shows a smaller yet noticeable impact on growth. Water activity measures the availability of free water for microbial activities, and since *Pseudomonas* spp. require moisture to thrive, maintaining low water activity can help inhibit their growth. Most foods have water activities high enough to support microbial growth; however, controlling this variable can be an effective measure in reducing growth rates. Finally, pH has the least impact among the factors. While pH affects microbial growth by influencing enzyme stability and nutrient availability, *Pseudomonas* spp. can tolerate a range of pH levels, especially near neutrality, which may explain its lower importance relative to the other factors. However, maintaining pH levels outside of this range can still contribute to controlling growth, although it is less effective compared to controlling time, temperature or water activity.

**Figure 6:**
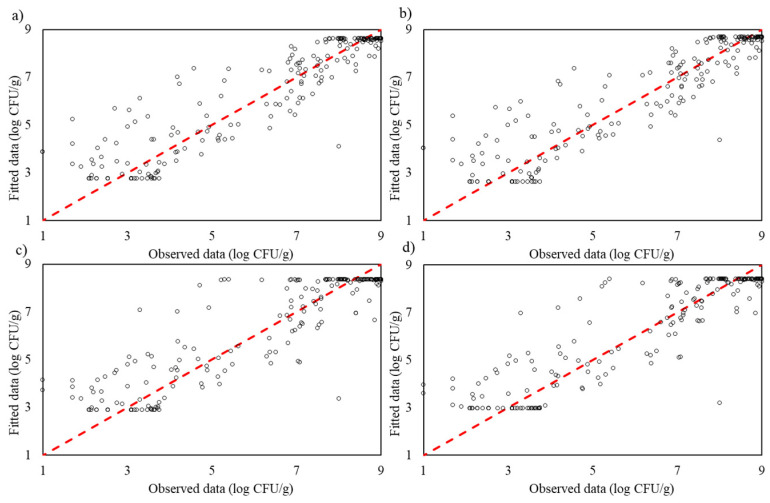
The observed and predicted *Pseudomonas* spp. in culture medium using traditional models: (**a**) modified Gompertz, (**b**) Logistic, (**c**) Baranyi and (**d**) Huang for testing process.

The test data’s performance for the traditional modelling approaches—Gompertz, Logistic, Baranyi and Huang models—shows varying levels of predictive accuracy. The Gompertz model captures the general trend but exhibits noticeable deviations from the ideal line, indicating limited precision. The Logistic model shows slightly better alignment with the ideal line, suggesting improved accuracy in capturing growth dynamics, though still with some inconsistencies. The Baranyi model has the widest spread from the ideal line, reflecting lower predictive accuracy despite accounting for a lag phase, suggesting it struggles with the complexity of the growth data. Among the traditional models, the Huang model shows the closest fit to the ideal line, indicating the highest predictive accuracy and flexibility in modelling nonlinear growth trends (Figure 6).

**Figure 7:**
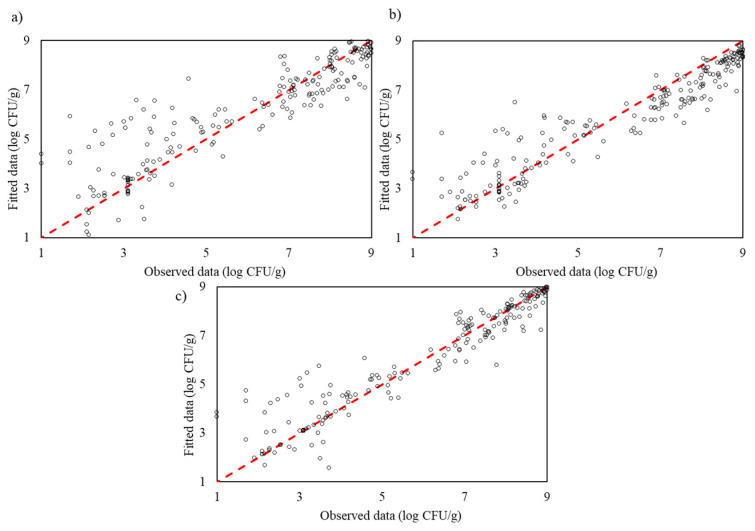
The observed and predicted *Pseudomonas* spp. in culture medium using machine learning models: (**a**) Support Vector Regression, (**b**) Random Forest Regression and (**c**) Gaussian Process Regression for testing process.

The test data’s performance of the machine learning models—Support Vector Regression, Random Forest Regression and Gaussian Process Regression—demonstrates their superior predictive accuracy and adaptability in capturing microbial growth dynamics. The SVR plot shows a relatively close alignment with the ideal line, though some minor deviations indicate that it may be sensitive to tuning parameters, especially in nonlinear regions. The RFR plot aligns more closely with the ideal line than SVR, illustrating its ensemble approach’s effectiveness in capturing complex interactions within the data, though it still shows slight scattering. GPR, however, displays the closest fit to the ideal line among the machine learning models, showcasing excellent alignment with minimal deviations. This indicates that GPR provides the highest predictive accuracy, capturing intricate patterns with greater robustness compared to SVR and RFR. These results underscore that machine learning models, particularly GPR, are more effective than traditional models in accurately modelling complex microbial growth patterns (Figure 7).

In comparing traditional modelling approaches with machine learning approaches for microbial growth prediction, it is evident that machine learning models generally provide enhanced accuracy and flexibility in handling complex growth dynamics. Traditional models, including Gompertz, Logistic, Baranyi, and Huang, are well-established and provide relatively straightforward interpretations due to their defined parametric structures, which are ideal for standard microbial growth patterns. Among these, the Gompertz model performs best, achieving an R^2^_adj_ of 0.861 and an RMSE of 0.007, closely followed by the Logistic model (Table 3). These models maintain decent accuracy (highest for the Huang model at 69.8%) and offer good predictive bias (*B*_f_) and accuracy factor (*A*_f_) scores. However, their limited adaptability to nonlinear and non-standard growth patterns restricts their performance in more complex scenarios, as indicated by their lower accuracy values compared to machine learning models.

Machine learning models, including Support Vector Regression, Random Forest Regression and Gaussian Process Regression, demonstrate superior capability by not assuming a predefined functional form, allowing them to capture intricate, nonlinear growth behaviours effectively. GPR, in particular, stands out with the highest R^2^_adj_ of 0.923 and the lowest RMSE of 0.005, showcasing its robustness and reliability in handling complex data patterns. Its accuracy (84.3%) surpasses all other models, traditional and machine learning alike. RFR also performs notably well, with an R^2^_adj_ of 0.884 and an RMSE of 0.006, benefiting from its ensemble approach to account for variable interactions. SVR, while effective with an R^2^_adj_ of 0.834, shows limitations when compared to GPR and RFR, potentially due to sensitivity in high-dimensional spaces and the need for careful parameter tuning. Furthermore, it is important to note that the R^2^_adj_ of the SVR is slightly lower than that of traditional models, except for the Branyi model. In terms of the bias factor (*B*_f_) and accuracy factor (*A*_f_), machine learning models generally display a closer alignment to ideal values, with RFR having a nearly perfect *B*_f_ of 0.998 and GPR achieving the lowest *A*_f_ (1.100), indicating greater consistency and reliability. All these results affirm that while traditional models offer interpretable and moderately accurate predictions suitable for simpler growth dynamics, RFR and GPR provide a higher degree of predictive power, accuracy and flexibility, making them better suited for complex and nonlinear microbial growth modelling scenarios.

**Figure 8:**
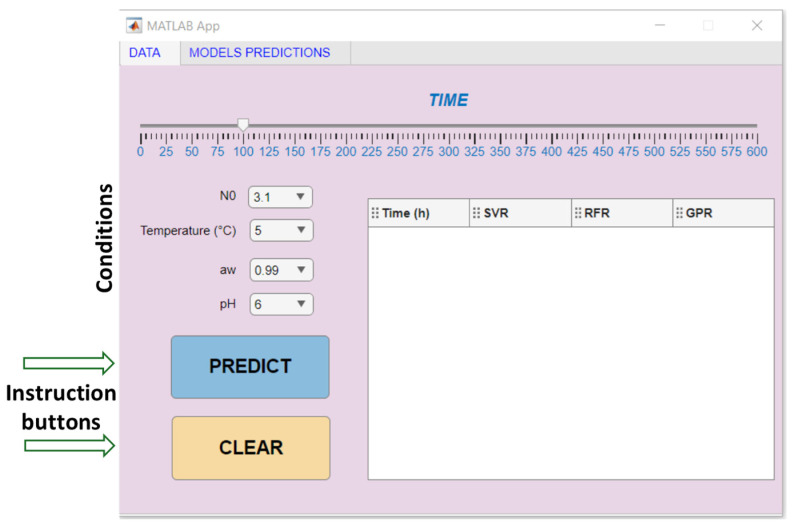
Illustration of developed software and its parts.

The machine learning models developed for predicting microorganism growth were integrated into a user-friendly software interface, allowing users to easily input parameters and visualize predicted microbial counts. This interface, illustrated in Figure 8, showcases a streamlined design aimed at simplifying the prediction process, making it accessible even to users without extensive technical knowledge. Key components of the interface include input fields for essential parameters such as temperature, pH, water activity, and other relevant environmental factors. Upon entering these values, the software instantly generates predictions using trained machine learning models like GPR, RFR and SVR, providing outputs on microbial growth rates and expected counts. In addition, this software has been made accessible to a broader audience via the GitHub platform. The repository, located under the name “ftarlak/Pseu_Calculator”, includes not only the code but also a brief video tutorial that guides users through the installation and usage steps. This video demonstration helps users understand the functionality of each component within the interface, from data entry to interpreting prediction outputs.

## 4. Conclusions

The comparative analysis of traditional and machine learning models for microbial growth prediction underscores the distinct advantages and limitations of each approach. Traditional models like Gompertz, Logistic, Baranyi and Huang are valued for their interpretability and ability to capture standard S-shaped growth patterns, but they struggle with complex, nonlinear dynamics due to their fixed parametric structures. Among these, the Huang and Gompertz models showed relatively stronger performance, yet their predictive power was outperformed by machine learning models. Machine learning approaches demonstrated superior adaptability to complex data patterns. GPR emerged as the most robust, achieving an R^2^_adj_ of 0.923 and RMSE of 0.005, making it highly effective for capturing nonlinear growth behaviours. RFR also demonstrated strong performance, achieving an R^2^_adj_ of 0.884 and an RMSE of 0.006, while SVR was somewhat constrained by its sensitivity to high-dimensional data and noise. Overall, while traditional models are useful for simpler, interpretable predictions, machine learning models, particularly GPR, offer enhanced accuracy and adaptability, making them better suited for complex, dynamic data environments. This analysis highlights the potential of machine learning as a robust tool for microbial growth prediction and suggests that future research could benefit from hybrid approaches that balance the interpretability of traditional models with the analytical power of machine learning.

## Figures and Tables

**Table 1:** Correlation values between main predictor variables (temperature (°C), water activity and pH).

Predictors	Temperature (°C)	Water Activity	pH
Temperature (°C)	1	−0.048	0.098
Water activity	−0.048	1	−0.164
pH	0.098	−0.164	1

**Table 2:** Evaluation indices of traditional and machine learning modelling approaches for training process.

Approach	Traditional Modelling Approach				Machine Learning Modelling Approach		
Model	Gompertz	Logistic	Baranyi	Huang	SVR	RFR	GPR
R^2^_adj_	0.813	0.844	0.790	0.850	0.854	0.893	0.959
RMSE	0.022	0.020	0.023	0.020	0.019	0.017	0.010

**Table 3:** Evaluation indices of traditional and machine learning modelling approaches for testing process.

Approach	Traditional Modelling Approach				Machine Learning Modelling Approach		
Model	Gompertz	Logistic	Baranyi	Huang	SVR	RFR	GPR
R^2^_adj_	0.861	0.858	0.818	0.836	0.834	0.884	0.923
RMSE	0.007	0.007	0.008	0.007	0.007	0.006	0.005
*B*_f_	1.032	1.030	1.035	1.036	1.047	0.998	1.031
*A*_f_	1.157	1.159	1.173	1.162	1.167	1.138	1.100
Accuracy	0.682	0.690	0.649	0.698	0.740	0.686	0.843

## Data Availability

The original contributions presented in this study are included in the article/Supplementary Materials. Further inquiries can be directed to the author.

## Supplementary Materials

The following supporting information can be downloaded at: https://www.mdpi.com/article/10.3390/life14111490/s1, Video S1: Guide video of the developed software.
